# Wandering Spleen After Sleeve Gastrectomy as a Cause of Sigmoid Volvulus

**DOI:** 10.7759/cureus.50447

**Published:** 2023-12-13

**Authors:** Elise N Snyder, Aniruddha Rao, Scott T Rehrig

**Affiliations:** 1 Department of Surgery, Anne Arundel Medical Center, Annapolis, USA; 2 School of Medicine, University of Maryland School of Medicine, Baltimore, USA

**Keywords:** general emergency surgery, emergency general surgery, differential diagnoses of acute abdomen, sigmoid volvulus, wandering spleen, bariatric surgery complications

## Abstract

The report highlights a rare instance of colonic volvulus due to a wandering spleen. Wandering spleen is characterized by the displacement of the spleen due to absent or weakened ligaments due to congenital factors or acquired factors such as pregnancy or prior surgery leading to ligament disruption. The 26-year-old patient presented with severe abdominal pain and distention, leading to a diagnosis of sigmoid volvulus secondary to the wandering spleen. This case underscores the importance of considering the wandering spleen in the differential diagnosis of acute abdomen, especially in patients with a surgical history of gastric sleeve resection. The article emphasizes the critical role of imaging in diagnosis and the necessity of timely surgical intervention to prevent severe complications. The case contributes to a broader understanding of the wandering spleen, particularly in post-surgical contexts, highlighting diagnostic challenges and management strategies.

## Introduction

Wandering spleen is an exceedingly rare condition caused by a weakening or breakdown in ligaments holding the spleen in its normal anatomic position in the left upper quadrant of the abdomen [1,2]. The condition may be congenital through an inborn laxity of the splenic ligaments, possibly associated with prior parity [3]. Diagnosing a wandering spleen is challenging, as the abnormally located spleen is often confused with an abdominal mass. The condition is often diagnosed only upon symptom onset following torsion of the splenic pedicle and compromise of splenic blood supply, constituting a surgical emergency [1]. When the spleen is viable, other surgeons have performed splenopexy, but if non-viable, splenectomy is required [4].

Estimates of prevalence are low at 0.2% [4]. Although the condition is rare, it is important to understand its existence and maintain it in a differential diagnosis of acute abdomen to identify the condition quickly and prevent significant morbidity and mortality. Here, we present a case of a female patient with a prior history of sleeve gastrectomy who suddenly and rapidly developed diffuse abdominal pain. In this complex presentation, in addition to splenic ischemia, the patient was found to have sigmoid volvulus due to torsion of redundant sigmoid around the splenic pedicle.

## Case presentation

This is the case of a 26-year-old nulliparous female with a past surgical history of sleeve gastrectomy and paraesophageal hernia repair in March 2022 who presented in April 2023 with progressive abdominal pain, intermittent nausea, and non-bilious emesis for two days. Her last bowel movement was on the morning of her presentation to the emergency department. The remainder of the history was non-contributory. On physical examination, she was ill-appearing. Her abdomen was distended with severe tenderness to palpation in the right lower quadrant with rebound tenderness throughout.

**Figure 1:**
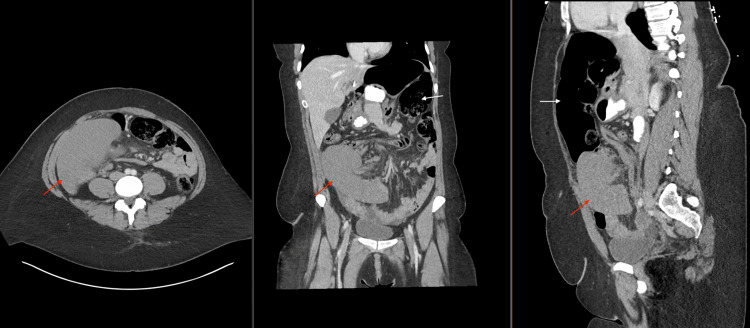
Preoperative CT scan images No free air is seen, and the ischemic spleen is in the right lower quadrant (red arrows). The dilatated and volvulized left colon is shown by white arrows.

We obtained laboratory studies and a multiaxial contrast-enhanced CT scan. Laboratory results were within normal limits. CT findings demonstrated no free air and an ischemic spleen in the right lower quadrant (Figure 1). CT further noted marked sigmoid dilatation with mesenteric edema. 

Due to the examination noting peritoneal signs and imaging findings concerning for splenic ischemia and sigmoid volvulus, we proceeded with exploratory laparotomy. The imaging findings suggested multi-visceral involvement in the volvulus, therefore the operative team chose an open approach.  

**Figure 2:**
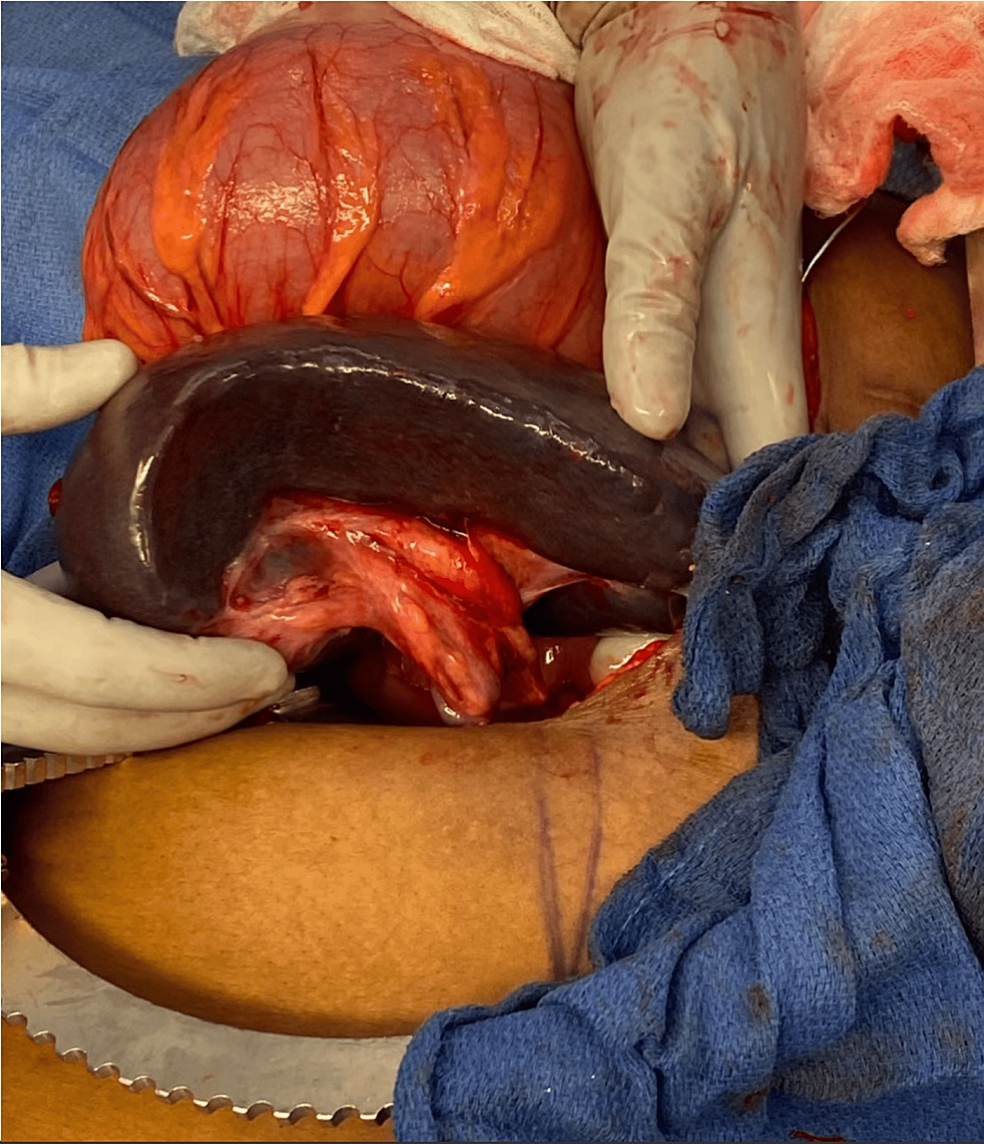
Ischemic spleen and hyperemic sigmoid volvulus

Intraoperatively, the sigmoid colon was volvulized around the elongated splenic pedicle. The splenic vessels were torsed and the spleen was noted to be ischemic. The sigmoid colon was distended and hyperemic (Figure 2). The sigmoid mesentery and parenchyma had stigmata of chronic volvulus. Once the sigmoid was detorsed, the spleen was easily mobilized for splenectomy without significant adhesions. The splenic hilum was divided with suture ligatures and sent off the field. We then proceeded with sigmoidectomy. The sigmoid was divided proximally and distally out of the region of hyperemia with intestinal staplers. We established intestinal continuity via an isoperistaltic stapled colorectal anastomosis.

**Figure 3:**
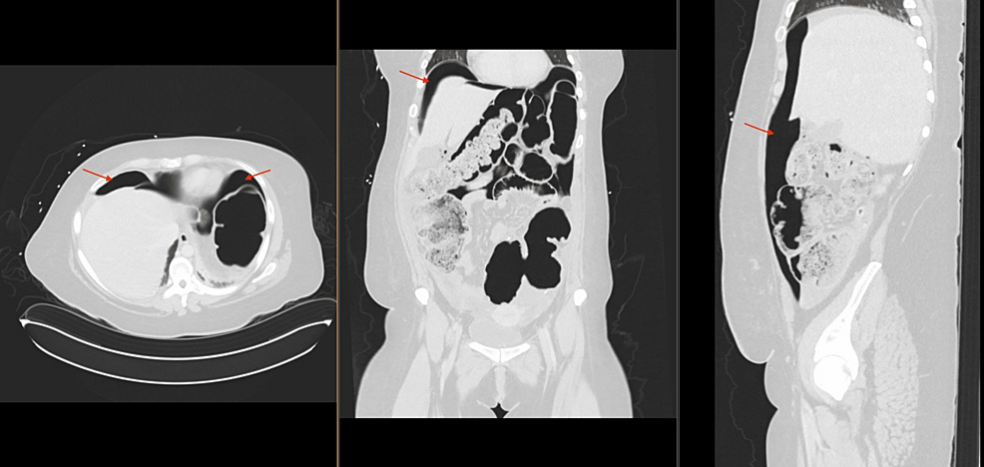
CT images postoperative day eight Large volume free air anastomotic leak (red arrows).

The patient recovered as expected until postoperative day eight when she developed new leukocytosis of 23 thousand, abdominal pain, and distention. A CT scan of the abdomen and pelvis noted findings of free air and fluid concerning for an anastomotic leak (Figure 3). The patient underwent an unplanned return to the operating room.

**Figure 4:**
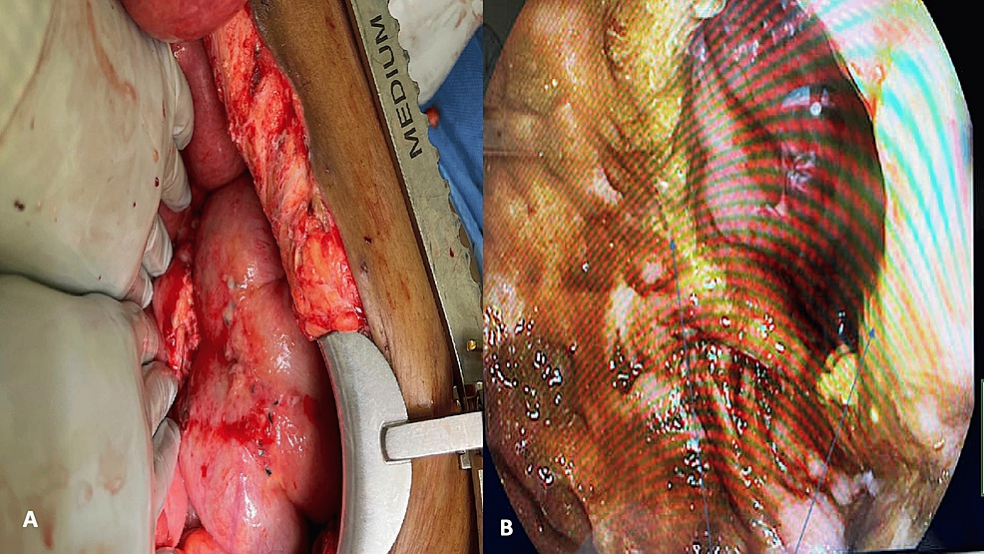
Intraoperative images (4A) External inspection of colorectal anastomosis noting no evidence of leak via endoscopy leak test; (4B) Endoscopic view noting viable anastomosis, arrows demarcating staple line.

Intraoperatively, there was purulent fluid concerning an anastomotic leak; however, the anastomosis appeared intact. There was no other perforation identified on exploration to explain her symptoms and imaging findings. The operative team performed an endoscopic leak test, noting no evidence of anastomotic failure (Figures 4A, 4B). The patient likely had a sealed anastomotic leak. Thereafter, the patient recovered as expected. She was discharged home on postoperative day fourteen. At the time of this report, eight months after discharge, the patient is in good health with no further hospitalizations.

## Discussion

The causes of wandering spleens are unclear. It is hypothesized that failure of certain critical events during embryonic development may lead to defective development of the splenic ligaments, resulting in congenital wandering spleen [5]. Acquired wandering spleen is associated with women of childbearing age, prompting the theory that hormonal changes may contribute to the laxity of splenic ligaments [4]. In contrast, surgeons have reported other underlying conditions causing splenomegaly, such as malaria or mononucleosis [1,4]. The incidence appears bimodal, with an increase in early childhood (especially within the first year of life) and an increase in the third and fourth decade of life, most frequently in females of childbearing age [4]. Regardless of ultimate etiology, it appears that the congenital or acquired absence of splenic supports allows the spleen to wander freely, leading to a high risk of vascular pedicle volvulus. As observed in our case, the wandering spleen may cause concomitant injury to other abdominal organs, complicating the surgical management.

In addition to this case, other authors have reported a wandering spleen following sleeve gastrectomy in the literature [6]. The dissection of the short gastric and gastrosplenic ligament during sleeve gastrectomy may lead to a wandering spleen in a patient with laxity at other splenic attachments. There have also been reports of acquired wandering spleen following operations with similar dissections, such as Nissen fundoplication, supporting the notion that there may be iatrogenic causes of wandering spleen [7]. This patient also had a paraesophageal hernia repair at the time of her sleeve gastrectomy. We did not find any case reports illustrating wandering spleen post paraesophageal hernia repair, but there are reports of wandering spleen after congenital diaphragmatic hernia repair [8]. Further, there are reports of wandering spleen causing both gastric and small intestinal volvulus, illustrating the potential for wandering spleen to cause secondary pathology in addition to splenic ischemia [8,9].

While the wandering spleen is rare, surgeons should consider it as part of their differential diagnosis in cases of acute abdomen, especially in females ages 20 to 40, particularly those who have undergone sleeve gastrectomy or other procedures involving dissection of the gastrosplenic ligament [7,10]. Diagnostic evaluation begins with a history, which may reveal non-specific, vague, intermittent abdominal pain, possibly representing intermittent torsion of the spleen [3,4,11]. Physical examination may reveal a large, painful, mobile abdominal mass [1,3,11]. Other clinical signs may include diffuse abdominal tenderness, rebound, guarding, or even hemodynamic instability, all thought to be related to torsion of the spleen around its blood supply. Further evaluation includes imaging studies to establish the diagnosis definitively. Laboratory studies generally are non-specific and tend to be less helpful. Point of care abdominal ultrasound may be used to highlight an ectopic location of the spleen; however, definitive evaluation requires a CT scan with intravenous contrast [3]. An alternative method for assessing splenic vascular compromise is with a Doppler ultrasound.  

In this case, we opted for urgent surgical intervention as the blood supply to the spleen appeared compromised on imaging. Other authors have described splenic salvage via splenopexy in less urgent clinical situations. However, if the spleen is beyond vascular recovery, the best treatment approach is splenectomy [12]. We agree with other authors that it is not advisable to defer surgical management of a wandering spleen, even if asymptomatic, as up to 65% may eventually result in ischemia [13].

This case highlights a rare presentation of wandering spleen after sleeve gastrectomy. Sleeve gastrectomy is the most commonly performed bariatric surgery [14]. Although wandering spleen is rare, we have presented other cases in the literature of iatrogenic wandering spleen from surgical dissection of the stomach. Further research is needed to determine the frequency of wandering spleen after sleeve gastrectomy and if there is any advantage to surgical techniques to restore normal anatomy, such as omentopexy during bariatric surgery.

## Conclusions

A wandering spleen is an infrequent but potentially very dangerous clinical entity where the spleen detaches from its normal supporting ligaments and hangs precariously by its blood supply. We present an unusual case of sigmoid volvulus secondary to a wandering spleen. Surgical intervention can prevent splenectomy and avoid additional intestinal complications when diagnosed before compromise of the splenic vasculature. This is difficult as patients without acute complications from wandering spleen are largely asymptomatic. In patients presenting with an acute abdomen, especially women of childbearing age with a history of surgery requiring dissection of the greater curvature of the stomach from its splenic attachments, the wandering spleen should remain on the differential. In addition, sleeve gastrectomy is the most commonly performed bariatric procedure worldwide. Although a wandering spleen is still a relatively rare diagnosis, with the increase in sleeve gastrectomy, we may see a concurrent increase in wandering spleen, further stressing the importance of recognizing this rare presentation.
